# A non-canonical landscape of the microRNA system

**DOI:** 10.3389/fgene.2014.00337

**Published:** 2014-09-23

**Authors:** Gabriel A. Cipolla

**Affiliations:** Laboratory of Human Molecular Genetics, Department of Genetics, Federal University of ParanáCuritiba, Brazil

**Keywords:** miRNA, non-canonical, miRNA*, subcellular localization, 5′UTR, coding sequence, RNA activation, transcriptional silencing

## Abstract

Microribonucleic acids, best known as microRNAs or miRNAs, are small, non-coding RNAs with important regulatory roles in eukaryotic cells. Here, I present a broad review on highly relevant but generally non-depicted features of miRNAs, among which stand out the non-conventional miRNA seed sites, the unusual messenger RNA (mRNA) target regions, the non-canonical miRNA-guided mechanisms of gene expression regulation, and the recently identified new class of miRNA ligands. Furthermore, I address the miRNA uncommon genomic location, transcription, and subcellular localization. Altogether, these unusual features and roles place the miRNA system as a very diverse, complex, and intriguing biological mechanism.

## INTRODUCTION

MicroRNAs (miRNAs), a class of non-coding RNAs (ncRNAs) of approximately 23 nucleotides (nt), are well known for their role in RNA interference (RNAi), where they mediate posttranscriptional gene expression regulation (Bartel, 2009). miRNA genes generally reside in intergenic regions carrying their own promoters or in intronic regions of transcription units, therefore sharing the host gene promoter and being commonly transcribed by RNA polymerase (Pol) II (Bartel, 2004; Cai et al., 2004; Lee et al., 2004; Kim and Nam, 2006). The approximately 1000 nt primary transcript (pri-miRNA), a stem-loop structure with long single-stranded ends, is typically processed by a nuclear RNase III enzyme named Drosha, giving rise to an approximately 60 nt hairpin intermediate known as the miRNA precursor (pre-miRNA). This pre-miRNA bears an approximately 2 nt overhang at the 3′-end of the stem-loop structure, which is important for its further processing in the cytoplasm by a second RNase III enzyme, named Dicer. This second cleavage removes the loop of the pre-miRNA, leaving another 2 nt 3′overhang and, thus, a miRNA duplex (Bartel, 2004; Cullen, 2004). In general, only one strand of the miRNA duplex is loaded into the RNA-induced silencing complex (RISC), where it will guide, in a sequence-specific manner, mRNA degradation or translation inhibition (Cullen, 2004; Bartel, 2009). This interaction is mainly reported to take place between the 5′-end of the miRNA and the 3′untranslated region (UTR) of the mRNA. The process described above is generally seen as the miRNA canonical aspects and their main mode of action. However, current literature has brought to light non-conventional miRNA features, which are the center of this review.

**FIGURE 1:**
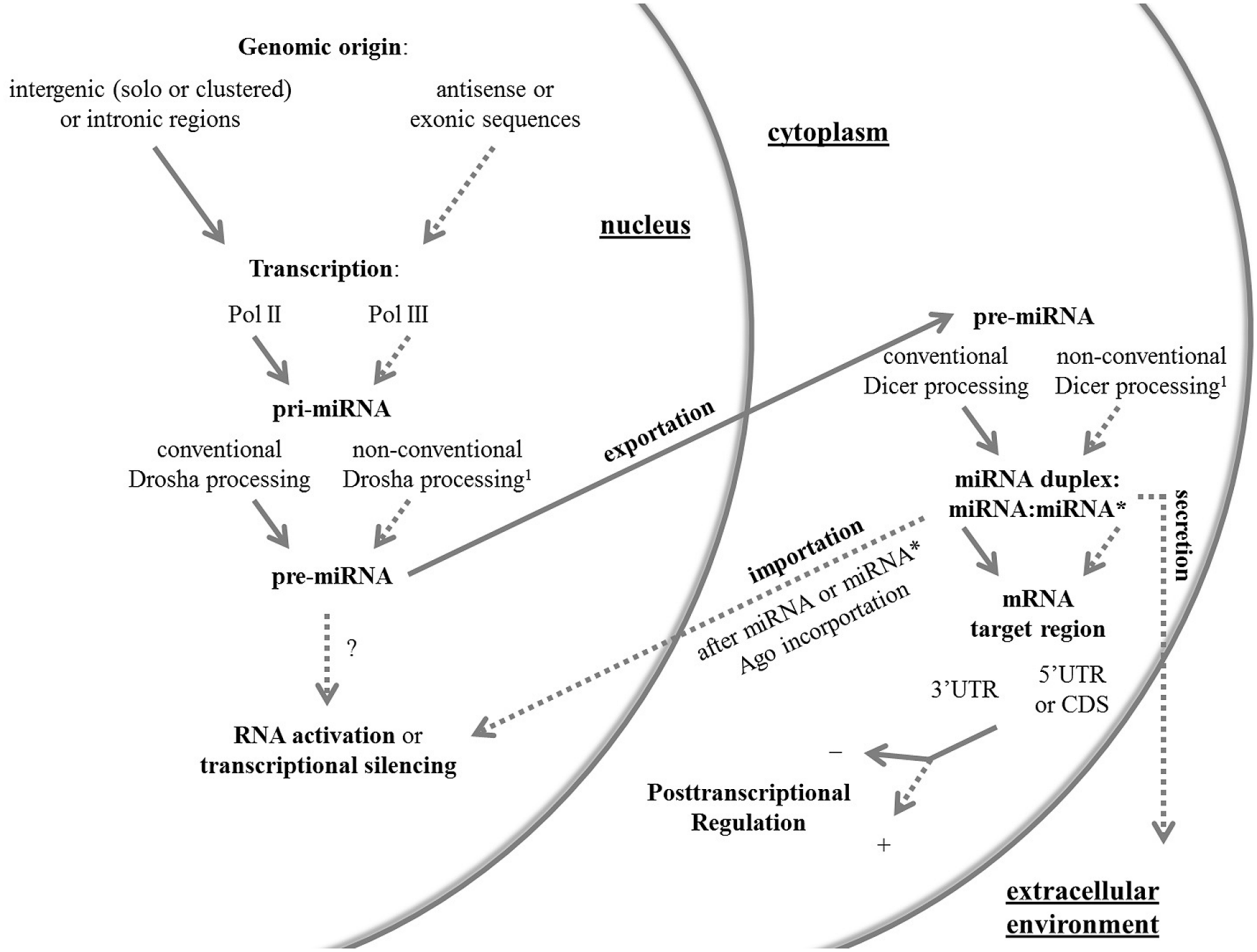
**An overview of the canonical and non-canonical aspects of miRNAs.** Conventional and non-conventional features of the miRNA system in animals are outlined by full and dashed arrows, respectively. The non-conventional route can be represented by one or more unusual aspects. ^1^ Non-conventional Drosha and Dicer pathways have been reviewed elsewhere (Yang and Lai, 2011; Ha and Kim, 2014). Ago, Argonaute protein; CDS, coding sequence; Pol, RNA polymerase; pre-miRNA, precursor miRNA; pri-miRNA, primary miRNA; UTR, untranslated region.

Hereafter, I review the current knowledge in the miRNA field with focus on the non-canonical facets of these molecules in animals, which are summarized in **Figure 1**. For a complete review on non-conventional Drosha and Dicer pathways, readers can address two published articles (Yang and Lai, 2011; Ha and Kim, 2014).

## UNUSUAL microRNA GENOMIC LOCATION AND TRANSCRIPTION

Although there is no consensus about the most frequent miRNA genomic location in animals due, among other factors, to varying miRNA genomic distribution in different species, there is no doubt that they mainly map to intergenic regions as solo or clustered genes or to intronic regions of defined protein-coding or non-coding transcription units (Bartel, 2004; Kim and Nam, 2006). The most striking deviation was shown in mouse testes, where approximately 30% of the miRNA genes mapped to exonic sequences (Ro et al., 2007).

While it has been assumed that intronic miRNAs rely on host gene transcription in order to be expressed (Bartel, 2004), compelling evidence has suggested that a set of intronic miRNAs can be expressed, both in humans and *Caenorhabditis elegans*, independently of their host gene (Isik et al., 2010; Monteys et al., 2010). In a *Drosophila melanogaster* short RNA data meta-analyses, Berezikov et al. (2011) identified 12 miRNA loci also giving rise to miRNAs from antisense strand transcription and processing. Besides this potential source of novel miRNAs in *Drosophila*, Berezikov et al. (2011) located miRNA loci in coding sequences and UTRs. Antisense strand origin was consistently found in humans, although miRNAs derived from the antisense transcription of the miRNA loci showed to be lowly expressed (Burroughs et al., 2011).

Canonical transcription of miRNAs involves Pol II, the same enzyme generally producing mRNAs (Cai et al., 2004; Lee et al., 2004). However, a miRNA cluster in human chromosome 19 (C19MC) downstream from an Alu repeat was shown to be associated only with Pol III, suggesting that this same enzyme, responsible for Alu activity, is also involved in C19MC transcription (Borchert et al., 2006). Moreover, on a bioinformatic approach, the authors proposed that miRNAs flanked upstream by tRNA-, besides Alu- based promoters, might be transcribed by Pol III. However, the first findings were challenged by Bortolin-Cavaillé et al. (2009), who proposed that C19MC miRNAs are actually derived from Pol II placenta-specific non-protein-coding transcripts. Still, further studies have shown that miRNAs of murine γ-herpesvirus 68 (Bogerd et al., 2010) and herpesvirus 4 (Diebel et al., 2010), and miRNA-like small RNAs of *Neurospora crassa* (Yang et al., 2013) are indeed transcribed by Pol III. Therefore, it remains to be demonstrated whether functional miRNAs can be produced through Pol III activity in animals.

## A NOT SO PASSENGER STRAND

It has long been proposed, since the initial studies of miRNAs in *C. elegans* (Lim et al., 2003), *D. melanogaster* (Aravin et al., 2003), and mouse (Lagos-Quintana et al., 2002), that during miRNA biogenesis and maturation only one of the arms of the pre-miRNA fold-back is generally found accumulating at steady levels. The less common mature miRNA sequence derived from the precursor has been named miRNA^∗^ (miRNA star) or “passenger strand,” while the most abundant one has been named miRNA or “guide strand,” as it is thought to be preferentially incorporated into Argonaute (Ago) protein complexes and, therefore, to guide posttranscriptional regulation (Lau et al., 2001; Yang et al., 2011).

However, several studies have suggested that miRNA^∗^ strands are more abundant than initially thought and that these might be more than mere carriers of the guide strand. Comparisons between human, chimpanzee, mouse, rat, dog, and chicken genomes revealed that several human miRNA^∗^ strands are highly conserved, especially at the seed sequence vicinity, which, in turn, exhibits significant 3′UTR complementarity across vertebrate evolution (Yang et al., 2011). miRNA^∗^ seed sequences and center regions have also been shown to be conserved across Drosophilid evolution (Okamura et al., 2008) and a group of typical vertebrates (human, zebrafish, chicken, and frog; Guo and Lu, 2010). By analyzing 10 different libraries from human and mouse deep-sequencing data, Kuchenbauer et al. (2011) found among all detected miRNAs a percentage of miRNA^∗^ ranging from 0.3 to 12.3%, suggesting a tissue and species-specific miRNA^∗^ expression. Moreover, classification into miRNA/miRNA^∗^ ratio groups pointed out that approximately 13% of all ratios favor the miRNA^∗^, while approximately 13, 24, and 50% favor the miRNA at low, intermediate, and high ratios, respectively. The class of miRNA duplexes giving rise to balanced strand expression was termed “β-duplexes,” while the class of miRNA duplexes giving rise to a dominant strand was called “α-duplexes” (Kuchenbauer et al., 2011). Finally, these broader and evolutionary analyses are accompanied by reporter assays focusing on validating the miRNA^∗^ functionality (Okamura et al., 2008; Ogata et al., 2010; Kuchenbauer et al., 2011; Yang et al., 2011; Byrd et al., 2012; Niederer et al., 2012; Chang et al., 2013; Goedeke et al., 2013; Martin et al., 2014). Altogether, these evidences suggest that all miRNA loci are potential dual-function genes, as two distinct miRNAs may originate from the same hairpin and, therefore, target different sets of genes (Okamura et al., 2008; Ogata et al., 2010; Ohanian et al., 2013).

## NUCLEUS-TO-CYTOPLASM microRNA TRANSPORT: A TWO-WAY ROUTE?

It is a general assumption that pre-miRNAs are transported by Exportin-5 in a one-way direction from nucleus to cytoplasm (Yi et al., 2003; Lund et al., 2004). However, many lines of evidence indicate that these molecules can also be guided back to the nucleus. A hexanucleotide terminal motif of miR-29b is responsible for this cytoplasm to nucleus transport (Hwang et al., 2007). CRM1 (Exportin-1), known to transport different classes of RNAs, enables the miRNA nuclear import (Castanotto et al., 2009). miRNAs and piwi-interacting RNAs (piRNAs) were found in the nuclei of spermatocytes and Sertoli cells (Marcon et al., 2008). These miRNAs may enter the nucleus to undergo modifications, associate with nuclear proteins or with target transcripts, participate in chromatin remodeling, or regulate ncRNAs. An example of miRNAs directly regulating transcriptional silencing includes the knockdown of POLR3D mRNA expression due to increased levels of mature miR-320 (Kim et al., 2008). Other examples of miRNAs found in the nucleus are miR-709, miR-690, miR-30e (Tang et al., 2012), and miR-122 (Földes-Papp et al., 2009). miRNAs can also be found in the nucleolus as precursor forms, like miR-494 and miR-664, and as mature miRNAs, like miR-21, miR-1, miR-351, miR-206 (Politz et al., 2006, 2009), and miR-320 (Marcon et al., 2008). Another intriguing subcellular localization of miRNAs is mitochondria, where they may modulate apoptosis processes in a coordinated way (Kren et al., 2009).

Ago family proteins have also been consistently detected inside the nucleus (Ohrt et al., 2008; Rüdel et al., 2008; Tan et al., 2009; Ahlenstiel et al., 2012). It has been shown that Importin 8, besides being required for efficient binding of Ago2 to target mRNAs, directs Ago proteins to the nucleus of human cells (Weinmann et al., 2009). Taken together, these data add more evidence for the important function of regulatory RNAs inside the nuclear compartment.

## SPATIAL PREFERENCE: BINDING ELSEWHERE IN THE mRNA (AND miRNA)

After translation initiation, interactions between miRNAs and mRNAs resulting in translational repression may occur beyond the well-defined 3′UTR target, possibly taking place at the 5′UTR and coding sequence of the mRNA (Lytle et al., 2007). Although there is still some debate about the mechanisms of repression induced by miRNAs binding to different regions of their targets (Lytle et al., 2007; Forman et al., 2008), several computational screenings have pointed to putative miRNA sites in coding regions and 5′UTRs (Stark et al., 2007; Forman et al., 2008; Lee et al., 2009; Forman and Coller, 2010; Schnall-Levin et al., 2010). Several screenings bring experimentally validated results from reporter assays mainly (Forman et al., 2008; Schnall-Levin et al., 2010), confirming the possible physical interaction of miRNAs with 5′UTRs and/or coding regions. Several other studies focusing on such reporter assays have also confirmed this non-conventional targeting (Tay et al., 2008; Qin et al., 2010; Schnall-Levin et al., 2011). It remains a challenge to identify the broadness of miRNA:mRNA interactions that take place elsewhere in the mRNA.

Interestingly, Lee et al. (2009) observed among the 5′UTR motifs a preferential interaction of these sites with the 3′-end of miRNAs, suggesting that different ends of a miRNA may bind to both UTRs of an mRNA. Another type of interaction has been proposed to take place mainly between nucleotides 4–14 or 5–15 of a miRNA and its targeting sites, with these being named as “miRNA centered sites” (Shin et al., 2010). Together, these data indicate mRNA non-conventional seed sites, i.e., sites recognized by regions other than the miRNA 5′seed sequence.

## BEYOND POSTTRANSCRIPTIONAL GENE SILENCING

The discovery of miRNA-mediated gene expression regulation at the posttranscriptional level has revolutionized molecular biology, bringing new avenues to the treatment of several diseases, such as cancer and viral infections. However, emerging new mechanisms of gene expression regulation through miRNA activity should be considered by researchers when it comes to data interpretation and application. miRNAs have been recently proposed to recognize and guide transcription factors (TFs) to their correct gene promoters (Korla et al., 2013). Based on earlier results that many pre-miRNAs carry binding sites for TFs (Piriyapongsa et al., 2011) and that miRNAs target TFs (Dannemann et al., 2012), Korla et al. (2013) hypothesized that miRNAs would act as a decoy for TFs, driving them to their correct gene promoters. In a computational approach, miRNA seed sequences have also been shown to match gene promoters in a frequency comparable to miRNA/3′UTR matches and some miRNA/promoter pairs exhibited unusual sequence complementarity (Younger et al., 2009). Together with their previously discussed nuclear accumulation, these findings suggest that miRNAs may have an important function in this cellular compartment.

Other studies have deeply investigated and reported miRNAs as negative gene transcription regulators (Kim et al., 2008; Younger and Corey, 2011). Another line of evidence has proposed that dsRNAs also regulate gene expression through a mechanism termed as RNA activation (RNAa), in opposition to RNAi. RNAa has been shown to take place in humans at the transcriptional level as a consequence of both sequence-specific promoter (Li et al., 2006; Place et al., 2008), and/or sequence-specific antisense transcript (Morris et al., 2008; Schwartz et al., 2008) targeting by dsRNAs. In the first case, dsRNAs promoted gene activation by targeting AT-rich promoter regions, while in the second case activation was achieved through chromatin structure changes. More recently, RNAa was also shown to occur in non-human primates, mouse, and rat (Huang et al., 2010, 2012).

Positive regulation of gene expression is also an emerging feature of miRNAs at the posttranscriptional level. The first study to verify that miRNAs can act upregulating translation suggested a model by which human miR-369-3 directs, in a sequence-specific manner, the association of Ago and fragile X mental retardation-related protein 1 to the AU-rich element of the tumor necrosis factor-α (TNFα) mRNA under starvation conditions, leading to increased TNFα translation efficiency (Vasudevan et al., 2007). Similar results were found later and demonstrated that miR-10a targets the 5′UTR of ribosomal protein (RP) mRNAs resulting in RP enhanced translation under amino acid starvation. As a consequence, indirect global protein synthesis also occurred through the aforementioned miRNA–mRNA interaction, since it leads to increased availability of the translation machinery (Ørom et al., 2008). Together, this data may suggest that positive or negative posttranscriptional regulation by miRNAs might take place depending on the physiological state of the cell or organism. Another example of translation stimulation by miRNAs has been speculated by Jopling et al. (2005) and confirmed and detailed by Henke et al. (2008): the liver-specific miR-122, with two complementary sites on the 5′UTR of *Hepatitis C Virus* genome, induced viral RNA translation by contributing to the association of ribosomes in a sequence-specific manner.

A miRNA–miRNA posttranscriptional regulation network has been postulated on the basis of the finding that primary mRNA-like ncRNAs in mice are significantly underexpressed in tissues where their putative targeting miRNA is expressed (Zhao et al., 2008). Indeed, such an example of miRNA–miRNA regulation has been recently demonstrated: miR-709 inhibits miR-15a/16-1 maturation by binding to its primary transcript in the nucleus (Tang et al., 2012). Similarly, in *C. elegans*, let-7 mature miRNA carries a complementary sequence to its own primary transcript, whereby the interaction, in the presence of Ago protein ALG-1, induces primary transcript processing (Zisoulis et al., 2012). More recently, long non coding RNAs (lncRNAs) were shown to be potential targets of miRNAs, revealing also a possible miRNA-lncRNA regulation network (Jalali et al., 2013). These exciting results may open new possibilities to restoration strategies of pathologically relevant altered miRNA expression patterns.

## OTHER LIGANDS, NEW FUNCTIONS

It has for long been a dogma that miRNAs loaded in RISCs bind to mRNAs through Watson and Crick base pairing. Interaction with other molecules, such as proteins, remained unknown until recently, when miRNAs were found to bind to and inhibit the activity of a class of RNA-binding proteins (RBP), named heterogeneous ribonucleoproteins (hnRNPs), in a RISC-independent manner, but yet in a sequence-specific interaction (Eiring et al., 2010). Similar findings by Balkhi et al. (2013) have proposed miR-29 as a decoy for another RBP, named human antigen R (HuR). In a later study, two miRNAs with extracellular expression – miR-21 and miR-29a – were shown to reach a different cell and bind to murine Toll-like receptor (TLR) 7 and human TLR8, both located inside endosomes (Fabbri et al., 2012). The authors also verified that the miRNA–TLR interaction is immunologically relevant, as typical cytokines upregulated upon TLR activation were augmented in murine and human cells expressing TLR7 and TLR8, respectively, after treatment with miR-21 and miR-29a, miRNAs typically secreted by tumor cells. Similar results were found for another miRNA in the context of the nervous system. Using a murine experimental model, the extracellular miRNA let-7b was shown to directly activate the TLR7, inducing neurodegeneration (Lehmann et al., 2012). These results are extremely relevant as the authors also found that let-7b is overexpressed in Alzheimer’s disease subjects in relation to healthy controls. Another role for let-7b in the nervous system has been recently described, proposing that this miRNA acts as a pain mediator through TLR7 and ion channel activation in nociceptor neurons (Park et al., 2014). Mir-21, in turn, has been recently reported to mediate cell death of murine myoblasts through TLR7 (He et al., 2014). In summary, the existence of another class of miRNA-binding molecules is suggestive of an even wider role of these ncRNAs in development and disease in animals.

## CONCLUDING REMARKS

The canonical field of miRNAs has not yet been fully challenged and the next years of miRNA research will continue to teach us how complex this system is. With the growing usage of next-generation sequencing methods in transcriptomics, the identification of new genomic sources of miRNAs must rely on careful inspection of deep sequencing data, as RNA degradation fragments may be easily read as small ncRNAs. Another issue in miRNA research is that algorithm-based websites for the identification of miRNA binding sites, which are among the most diverse and used bioinformatic tools, frequently overlook 5′UTRs and coding sequences as potential targets of miRNAs and rarely consider their non-seed sequences as being likely to induce posttranscriptional regulation. This limitation clearly brings a bias to miRNA studies and might be responsible for several data misinterpretations. Moreover, the fast-growing field of extracellular miRNAs may contribute not only to the elucidation of the mechanisms involved in cell–cell communication, but also to our knowledge of the repertoire of miRNA-binding molecules. Finally, if on one hand the different emerging modes of gene expression regulation involving miRNAs suggest caution to their clinical application, they might on the other hand open new avenues for therapy strategies.

## Conflict of Interest Statement

The author declares that the research was conducted in the absence of any commercial or financial relationships that could be construed as a potential conflict of interest.

## References

[B1] Ahlenstiel C. L. Lim H. G. Cooper D. A. Ishida T. Kelleher A. D. Suzuki K. (2012). Direct evidence of nuclear Argonaute distribution during transcriptional silencing links the actin cytoskeleton to nuclear RNAi machinery in human cells. *Nucleic Acids Res.* 40 1579–1595 10.1093/nar/gkr891 22064859 PMC3287199

[B2] Aravin A. A. Lagos-Quintana M. Yalcin A. Zavolan M. Marks D. Snyder B. (2003). The small RNA profile during *Drosophila melanogaster* development. *Dev. Cell* 5 337–350 10.1016/S1534-5807(03)00228-4 12919683

[B3] Balkhi M. Y. Iwenofu O. H. Bakkar N. Ladner K. J. Chandler D. S. Houghton P. J. (2013). miR-29 acts as a decoy in sarcomas to protect the tumor suppressor A20 mRNA from degradation by HuR. *Sci. Signal.* 6:ra63 10.1126/scisignal.2004177 PMC3885907 23901138

[B4] Bartel D. P. (2004). MicroRNAs: genomics, biogenesis, mechanism, and function. *Cell* 116 281–297 10.1016/S0092-8674(04)00045-5 14744438

[B5] Bartel D. P. (2009). MicroRNAs: target recognition and regulatory functions. *Cell* 136 215–233 10.1016/j.cell.2009.01.002 19167326 PMC3794896

[B6] Berezikov E. Robine N. Samsonova A. Westholm J. O. Naqvi A. Hung J. H. (2011). Deep annotation of *Drosophila melanogaster* microRNAs yields insights into their processing, modification, and emergence. *Genome Res.* 21 203–215 10.1101/gr.116657.110 21177969 PMC3032924

[B7] Bogerd H. P. Karnowski H. W. Cai X. Shin J. Pohlers M. Cullen B. R.(2010). A mammalian herpesvirus uses noncanonical expression and processing mechanisms to generate viral MicroRNAs. *Mol. Cell* 37 135–142 10.1016/j.molcel.2009.12.016 20129062 PMC2818755

[B8] Borchert G. M. Lanier W. Davidson B. L. (2006). RNA polymerase III transcribes human microRNAs. *Nat. Struct. Mol. Biol.* 13 1097–1101 10.1038/nsmb1167 17099701

[B9] Bortolin-Cavaillé M. L. Dance M. Weber M. Cavaillé J. (2009). C19MC microRNAs are processed from introns of large Pol-II, non-protein-coding transcripts. *Nucleic Acids Res.* 37 3464–3473 10.1093/nar/gkp205 19339516 PMC2691840

[B10] Burroughs A. M. Ando Y. de Hoon M. J. Tomaru Y. Suzuki H. Hayashizaki Y. (2011). Deep-sequencing of human argonaute-associated small RNAs provides insight into miRNA sorting and reveals argonaute association with RNA fragments of diverse origin. *RNA Biol.* 8 158–177 10.4161/rna.8.1.14300 21282978 PMC3127082

[B11] Byrd A. E. Aragon I. V. Brewer J. W. (2012). MicroRNA-30c-2* limits expression of proadaptive factor XBP1 in the unfolded protein response. *J. Cell Biol.* 196 689–698 10.1083/jcb.201201077 22431749 PMC3308703

[B12] Cai X. Hagedorn C. H. Cullen B. R. (2004). Human microRNAs are processed from capped, polyadenylated transcripts that can also function as mRNAs. *RNA* 10 1957–1966 10.1261/rna.7135204 15525708 PMC1370684

[B13] Castanotto D. Lingeman R. Riggs A. D. Rossi J. J. (2009). CRM1 mediates nuclear-cytoplasmic shuttling of mature microRNAs. *Proc. Natl. Acad. Sci. U.S.A.* 106 21655–21659 10.1073/pnas.0912384106 19955415 PMC2787469

[B14] Chang K. W. Kao S. Y. Wu Y. H. Tsai M. M. Tu H. F. Liu C. J. (2013). Passenger strand miRNA miR-31* regulates the phenotypes of oral cancer cells by targeting RhoA. *Oral Oncol.* 49 27–33 10.1016/j.oraloncology.2012.07.003 22854067

[B15] Cullen B. R. (2004). Transcription and processing of human microRNA precursors. *Mol. Cell* 16 861–865 10.1016/j.molcel.2004.12.002 15610730

[B16] Dannemann M. Prüfer K. Lizano E. Nickel B. Burbano H. A. Kelso J. (2012). Transcription factors are targeted by differentially expressed miRNAs in primates. *Genome Biol. Evol.* 4 552–564 10.1093/gbe/evs033 22454130 PMC3342879

[B17] Diebel K. W. Smith A. L. van Dyk L. F. (2010). Mature and functional viral miRNAs transcribed from novel RNA polymerase III promoters. *RNA* 16 170–185 10.1261/rna.1873910 19948768 PMC2802027

[B18] Eiring A. M. Harb J. G. Neviani P. Garton C. Oaks J. J. Spizzo R. (2010). miR-328 functions as an RNA decoy to modulate hnRNP E2 regulation of mRNA translation in leukemic blasts. *Cell* 140 652–665 10.1016/j.cell.2010.01.007 20211135 PMC2924756

[B19] Fabbri M. Paone A. Calore F. Galli R. Gaudio E. Santhanam R. (2012). MicroRNAs bind to Toll-like receptors to induce prometastatic inflammatory response. *Proc. Natl. Acad. Sci. U.S.A.* 109 E2110–E2116 10.1073/pnas.1209414109 22753494 PMC3412003

[B20] Földes-Papp Z. König K. Studier H. Bückle R. Breunig H. G. Uchugonova A. (2009). Trafficking of mature miRNA-122 into the nucleus of live liver cells. *Curr. Pharm. Biotechnol.* 10 569–578 10.2174/138920109789069332 19619125

[B21] Forman J. J. Coller H. A. (2010). The code within the code: microRNAs target coding regions. *Cell Cycle* 9 1533–1541 10.4161/cc.9.8.11202 20372064 PMC2936675

[B22] Forman J. J. Legesse-Miller A. Coller H. A. (2008). A search for conserved sequences in coding regions reveals that the let-7 microRNA targets Dicer within its coding sequence. *Proc. Natl. Acad. Sci. U.S.A.* 105 14879–14884 10.1073/pnas.0803230105 18812516 PMC2567461

[B23] Goedeke L. Vales-Lara F. M. Fenstermaker M. Cirera-Salinas D. Chamorro-Jorganes A. Ramírez C. M. (2013). A regulatory role for microRNA 33* in controlling lipid metabolism gene expression. *Mol. Cell. Biol.* 33 2339–2352 10.1128/MCB.01714-12 23547260 PMC3648071

[B24] Guo L. Lu Z. (2010). The fate of miRNA* strand through evolutionary analysis: implication for degradation as merely carrier strand or potential regulatory molecule? *PLoS ONE* 5:e11387 10.1371/journal.pone.0011387 PMC2894941 20613982

[B25] Ha M. Kim V. N. (2014). Regulation of microRNA biogenesis. *Nat. Rev. Mol. Cell Biol.* 15 509–524 10.1038/nrm3838 25027649

[B26] He W. A. Calore F. Londhe P. Canella A. Guttridge D. C. Croce C. M. (2014). Microvesicles containing miRNAs promote muscle cell death in cancer cachexia via TLR7. *Proc. Natl. Acad. Sci. U.S.A.* 111 4525–4529 10.1073/pnas.1402714111 24616506 PMC3970508

[B27] Henke J. I. Goergen D. Zheng J. Song Y. Schüttler C. G. Fehr C. (2008). microRNA-122 stimulates translation of *Hepatitis C Virus* RNA. *EMBO J.* 27 3300–3310 10.1038/emboj.2008.244 19020517 PMC2586803

[B28] Huang V. Place R. F. Portnoy V. Wang J. Qi Z. Jia Z. (2012). Upregulation of Cyclin B1 by miRNA and its implications in cancer. *Nucleic Acids Res.* 40 1695–1707 10.1093/nar/gkr934 22053081 PMC3287204

[B29] Huang V. Qin Y. Wang J. Wang X. Place R. F. Lin G. (2010). RNAa is conserved in mammalian cells. *PLoS ONE* 5:e8848 10.1371/journal.pone.0008848 PMC2809750 20107511

[B30] Hwang H. W. Wentzel E. A. Mendell J. T. (2007). A hexanucleotide element directs microRNA nuclear import. *Science* 315 97–100 10.1126/science.1136235 17204650

[B31] Isik M. Korswagen H. C. Berezikov E. (2010). Expression patterns of intronic microRNAs in *Caenorhabditis elegans*. *Silence* 1:5 10.1186/1758-907X-1-5 PMC2835999 20226079

[B32] Jalali S. Bhartiya D. Lalwani M. K. Sivasubbu S. Scaria V. (2013). Systematic transcriptome wide analysis of lncRNA-miRNA interactions. *PLoS ONE* 8:e53823 10.1371/journal.pone.0053823 PMC3566149 23405074

[B33] Jopling C. L. Yi M. Lancaster A. M. Lemon S. M. Sarnow P. (2005). Modulation of *Hepatitis C Virus* RNA abundance by a liver-specific MicroRNA. *Science* 309 1577–1581 10.1126/science.1113329 16141076

[B34] Kim D. H. Saetrom P. Snøve O. Jr. Rossi J. J. (2008). MicroRNA-directed transcriptional gene silencing in mammalian cells. *Proc. Natl. Acad. Sci. U.S.A.* 105 16230–16235 10.1073/pnas.0808830105 18852463 PMC2571020

[B35] Kim V. N. Nam J. W. (2006). Genomics of microRNA. *Trends Genet.* 22 165–173 10.1016/j.tig.2006.01.003 16446010

[B36] Korla K. Arrigo P. Mitra C. K. (2013). Promoters, toll like receptors and microRNAs: a strange association. *Indian J. Biochem. Biophys.* 50 169–176 23898479

[B37] Kren B. T. Wong P. Y. Sarver A. Zhang X. Zeng Y. Steer C. J. (2009). microRNAs identified in highly purified liver-derived mitochondria may play a role in apoptosis. *RNA Biol.* 6 65–72 10.4161/rna.6.1.7534 19106625 PMC3972804

[B38] Kuchenbauer F. Mah S. M. Heuser M. McPherson A. Rüschmann J. Rouhi A. (2011). Comprehensive analysis of mammalian miRNA* species and their role in myeloid cells. *Blood* 118 3350–3358 10.1182/blood-2010-10-312454 21628414

[B39] Lagos-Quintana M. Rauhut R. Yalcin A. Meyer J. Lendeckel W. Tuschl T. (2002). Identification of tissue-specific microRNAs from mouse. *Curr. Biol.* 12 735–739 10.1016/S0960-9822(02)00809-6 12007417

[B40] Lau N. C. Lim L. P. Weinstein E. G. Bartel D. P. (2001). An abundant class of tiny RNAs with probable regulatory roles in *Caenorhabditis elegans*. *Science* 294 858–862 10.1126/science.1065062 11679671

[B41] Lee I. Ajay S. S. Yook J. I. Kim H. S. Hong S. H. Kim N. H. (2009). New class of microRNA targets containing simultaneous 5′-UTR and 3′-UTR interaction sites. *Genome Res.* 19 1175–1183 10.1101/gr.089367.108 19336450 PMC2704433

[B42] Lee Y. Kim M. Han J. Yeom K. Lee S. Baek S. H. (2004). MicroRNA genes are transcribed by RNA polymerase II. *EMBO J.* 23 4051–4060 10.1038/sj.emboj.7600385 15372072 PMC524334

[B43] Lehmann S. M. Krüger C. Park B. Derkow K. Rosenberger K. Baumgart J. (2012). An unconventional role for miRNA: let-7 activates Toll-like receptor 7 and causes neurodegeneration. *Nat. Neurosci.* 15 827–835 10.1038/nn.3113 22610069

[B44] Li L. C. Okino S. T. Zhao H. Pookot D. Place R. F. Urakami S. (2006). Small dsRNAs induce transcriptional activation in human cells. *Proc. Natl. Acad. Sci. U.S.A.* 103 17337–17342 10.1073/pnas.0607015103 17085592 PMC1859931

[B45] Lim L. P. Lau N. C. Weinstein E. G. Abdelhakim A. Yekta S. Rhoades M. W. (2003). The microRNAs of *Caenorhabditis elegans*. *Genes Dev.* 17 991–1008 10.1101/gad.1074403 12672692 PMC196042

[B46] Lund E. Güttinger S. Calado A. Dahlberg J. E. Kutay U. (2004). Nuclear export of microRNA precursors. *Science* 303 95–98 10.1126/science.1090599 14631048

[B47] Lytle J. R. Yario T. A. Steitz J. A. (2007). Target mRNAs are repressed as efficiently by microRNA-binding sites in the 5′UTR as in the 3′UTR. *Proc. Natl. Acad. Sci. U.S.A.* 104 9667–9672 10.1073/pnas.0703820104 17535905 PMC1887587

[B48] Marcon E. Babak T. Chua G. Hughes T. Moens P. B. (2008). miRNA and piRNA localization in the male mammalian meiotic nucleus. *Chromosome Res.* 16 243–260 10.1007/s10577-007-1190-6 18204908

[B49] Martin E. C. Elliott S. Rhodes L. V. Antoon J. W. Fewell C. Zhu Y. (2014). Preferential star strand biogenesis of pre-miR-24-2 targets PKC-alpha and suppresses cell survival in MCF-7 breast cancer cells. *Mol. Carcinog.* 53 38–48 10.1002/mc.21946 22911661 PMC4030540

[B50] Monteys A. M. Spengler R. M. Wan J. Tecedor L. Lennox K. A. Xing Y. (2010). Structure and activity of putative intronic miRNA promoters. *RNA* 16 495–505 10.1261/rna.1731910 20075166 PMC2822915

[B51] Morris K. V. Santoso S. Turner A. M. Pastori C. Hawkins P. G. (2008). Bidirectional transcription directs both transcriptional gene activation and suppression in human cells. *PLoS Genet.* 4:e1000258 10.1371/journal.pgen.1000258 PMC2576438 19008947

[B52] Niederer F. Trenkmann M. Ospelt C. Karouzakis E. Neidhart M. Stanczyk J. (2012). Down-regulation of microRNA-34a* in rheumatoid arthritis synovial fibroblasts promotes apoptosis resistance. *Arthritis Rheum.* 64 1771–1779 10.1002/art.34334 22161761

[B53] Ogata A. Furukawa C. Sakurai K. Iba H. Kitade Y. Ueno Y. (2010). Biaryl modification of the 5’-terminus of one strand of a microRNA duplex induces strand specificity. *Bioorg. Med. Chem. Lett.* 20 7299–7302 10.1016/j.bmcl.2010.10.077 21067927

[B54] Ohanian M. Humphreys D. T. Anderson E. Preiss T. Fatkin D. (2013). A heterozygous variant in the human cardiac miR-133 gene, MIR133A2, alters miRNA duplex processing and strand abundance. *BMC Genet.* 14:18 10.1186/1471-2156-14-18 PMC3599331 23497314

[B55] Ohrt T. Mütze J. Staroske W. Weinmann L. Höck J. Crell K. (2008). Fluorescence correlation spectroscopy and fluorescence cross-correlation spectroscopy reveal the cytoplasmic origination of loaded nuclear RISC *in vivo* in human cells. *Nucleic Acids Res.* 36 6439–6449 10.1093/nar/gkn693 18842624 PMC2582625

[B56] Okamura K. Phillips M. D. Tyler D. M. Duan H. Chou Y. T. Lai E. C. (2008). The regulatory activity of microRNA* species has substantial influence on microRNA and 3′UTR evolution. *Nat. Struct. Mol. Biol.* 15 354–363 10.1038/nsmb.1409 18376413 PMC2698667

[B57] Ørom U. A. Nielsen F. C. Lund A. H. (2008). MicroRNA-10a binds the 5′UTR of ribosomal protein mRNAs and enhances their translation. *Mol. Cell* 30 460–471 10.1016/j.molcel.2008.05.001 18498749

[B58] Park C. K. Xu Z. Z. Berta T. Han Q. Chen G. Liu X. J. (2014). Extracellular microRNAs activate nociceptor neurons to elicit pain via TLR7 and TRPA1. *Neuron* 82 47–54 10.1016/j.neuron.2014.02.011 24698267 PMC3982230

[B59] Piriyapongsa J. Jordan I. K. Conley A. B. Ronan T. Smalheiser N. R. (2011). Transcription factor binding sites are highly enriched within microRNA precursor sequences. *Biol. Direct* 6:61 10.1186/1745-6150-6-61 PMC3240832 22136256

[B60] Place R. F. Li L. C. Pookot D. Noonan E. J. Dahiya R. (2008). MicroRNA-373 induces expression of genes with complementary promoter sequences. *Proc. Natl. Acad. Sci. U.S.A.* 105 1608–1613 10.1073/pnas.0707594105 18227514 PMC2234192

[B61] Politz J. C. Hogan E. M. Pederson T. (2009). MicroRNAs with a nucleolar location. *RNA* 15 1705–1715 10.1261/rna.1470409 19628621 PMC2743059

[B62] Politz J. C. Zhang F. Pederson T. (2006). MicroRNA-206 colocalizes with ribosome-rich regions in both the nucleolus and cytoplasm of rat myogenic cells. *Proc. Natl. Acad. Sci. U.S.A.* 103 18957–18962 10.1073/pnas.0609466103 17135348 PMC1748159

[B63] Qin W. Shi Y. Zhao B. Yao C. Jin L. Ma J. (2010). miR-24 regulates apoptosis by targeting the open reading frame (ORF) region of FAF1 in cancer cells. *PLoS ONE* 5:e9429 10.1371/journal.pone.0009429 PMC2828487 20195546

[B64] Ro S. Park C. Sanders K. M. McCarrey J. R. Yan W. (2007). Cloning and expression profiling of testis-expressed microRNAs. *Dev. Biol.* 311 592–602 10.1016/j.ydbio.2007.09.009 17936267 PMC2121622

[B65] Rüdel S. Flatley A. Weinmann L. Kremmer E. Meister G. (2008). A multifunctional human Argonaute2-specific monoclonal antibody. *RNA* 14 1244–1253 10.1261/rna.973808 18430891 PMC2390805

[B66] Schnall-Levin M. Rissland O. S. Johnston W. K. Perrimon N. Bartel D. P. Berger B. (2011). Unusually effective microRNA targeting within repeat-rich coding regions of mammalian mRNAs. *Genome Res.* 21 1395–1403 10.1101/gr.121210.111 21685129 PMC3166825

[B67] Schnall-Levin M. Zhao Y. Perrimon N. Berger B. (2010). Conserved microRNA targeting in *Drosophila* is as widespread in coding regions as in 3′UTRs. *Proc. Natl. Acad. Sci. U.S.A.* 107 15751–15756 10.1073/pnas.1006172107 20729470 PMC2936641

[B68] Schwartz J. C. Younger S. T. Nguyen N. B. Hardy D. B. Monia B. P. Corey D. R. (2008). Antisense transcripts are targets for activating small RNAs. *Nat. Struct. Mol. Biol.* 15 842–848 10.1038/nsmb.1444 18604220 PMC2574822

[B69] Shin C. Nam J. W. Farh K. K. Chiang H. R. Shkumatava A. Bartel D. P. (2010). Expanding the microRNA targeting code: functional sites with centered pairing. *Mol. Cell* 38 789–802 10.1016/j.molcel.2010.06.005 20620952 PMC2942757

[B70] Stark A. Lin M. F. Kheradpour P. Pedersen J. S. Parts L. Carlson J. W. (2007). Discovery of functional elements in 12 *Drosophila* genomes using evolutionary signatures. *Nature* 450 219–232 10.1038/nature06340 17994088 PMC2474711

[B71] Tan G. S. Garchow B. G. Liu X. Yeung J. Morris J. P. IV Cuellar T. L. (2009). Expanded RNA-binding activities of mammalian Argonaute 2. *Nucleic Acids Res.* 37 7533–7545 10.1093/nar/gkp812 19808937 PMC2794174

[B72] Tang R. Li L. Zhu D. Hou D. Cao T. Gu H. (2012). Mouse miRNA-709 directly regulates miRNA-15a/16-1 biogenesis at the posttranscriptional level in the nucleus: evidence for a microRNA hierarchy system. *Cell Res.* 22 504–515 10.1038/cr.2011.137 21862971 PMC3292299

[B73] Tay Y. Zhang J. Thomson A. M. Lim B. Rigoutsos I. (2008). MicroRNAs to Nanog, Oct4 and Sox2 coding regions modulate embryonic stem cell differentiation. *Nature* 455 1124–1128 10.1038/nature07299 18806776

[B74] Vasudevan S. Tong Y. Steitz J. A. (2007). Switching from repression to activation: microRNAs can up-regulate translation. *Science* 318 1931–1934 10.1126/science.1149460 18048652

[B75] Weinmann L. Höck J. Ivacevic T. Ohrt T. Mütze J. Schwille P. (2009). Importin 8 is a gene silencing factor that targets Argonaute proteins to distinct mRNAs. *Cell* 136 496–507 10.1016/j.cell.2008.12.023 19167051

[B76] Yang J. S. Lai E. C. (2011). Alternative miRNA biogenesis pathways and the interpretation of core miRNA pathway mutants. *Mol. Cell* 43 892–903 10.1016/j.molcel.2011.07.024 21925378 PMC3176435

[B77] Yang J. S. Phillips M. D. Betel D. Mu P. Ventura A. Siepel A. C. (2011). Widespread regulatory activity of vertebrate microRNA* species. *RNA* 17 312–326 10.1261/rna.2537911 21177881 PMC3022280

[B78] Yang Q. Li L. Xue Z. Ye Q. Zhang L. Li S. (2013). Transcription of the major *Neurospora crassa* microRNA-like small RNAs relies on RNA polymerase III. *PLoS Genet.* 9:e1003227 10.1371/journal.pgen.1003227 PMC3547838 23349642

[B79] Yi R. Qin Y. Macara I. G. Cullen B. R. (2003). Exportin-5 mediates the nuclear export of pre-microRNAs and short hairpin RNAs. *Genes Dev.* 17 3011–3016 10.1101/gad.1158803 14681208 PMC305252

[B80] Younger S. T. Corey D. R. (2011). Transcriptional gene silencing in mammalian cells by miRNA mimics that target gene promoters. *Nucleic Acids Res.* 39 5682–5691 10.1093/nar/gkr155 21427083 PMC3141263

[B81] Younger S. T. Pertsemlidis A. Corey D. R. (2009). Predicting potential miRNA target sites within gene promoters. *Bioorg. Med. Chem. Lett.* 19 3791–3794 10.1016/j.bmcl.2009.04.032 19423343 PMC2709707

[B82] Zhao Y. He S. Liu C. Ru S. Zhao H. Yang Z. (2008). MicroRNA regulation of messenger-like noncoding RNAs: a network of mutual microRNA control. *Trends Genet.* 24 323–327 10.1016/j.tig.2008.04.004 18514357

[B83] Zisoulis D. G. Kai Z. S. Chang R. K. Pasquinelli A. E. (2012). Autoregulation of microRNA biogenesis by let-7 and Argonaute. *Nature* 486 541–544 10.1038/nature11134 22722835 PMC3387326

